# Supplementary material for: Ecological risk assessment and source identification of heavy metal pollution in vegetable bases of Urumqi, China, using the positive matrix factorization (PMF) method
Source: PLoS One. 2020 Apr 13;15(4):e0230191. doi: 10.1371/journal.pone.0230191 (PMC7153853; doi:10.1371/journal.pone.0230191)
Supplement: S1 Table — (DOCX) [file pone.0230191.s001.docx]

| S1 Table. Descriptive statistics of soil heavy metals in different suburban farmlands (Unit: mg/kg) | | | | | | | | | |
| --- | --- | --- | --- | --- | --- | --- | --- | --- | --- |
| Region | Heavy metals | | | | | | | | Data sources |
|  | Zn | Cu | Ni | Pb | Hg | Cd | Cr | As |  |
| Urumqi City, Northwest China | 94.44 | 34.88 | 33.36 | 22.07 | 0.079 | 0.174 | 60.98 | 6.89 | In this study |
| Taihang Piedmont Plain, China | 21.22 | 69.96 | 25.04 | 18.8 | 0.08 | 0.15 | 57.77 | 6.16 | Yang et al., 2009 |
| Tabriz City, Iran | 98.27 | 101.25 | 38.73 | 10.56 |  | 1.61 | 87.4 | - | Taghipour et al., 2013 |
| Nanjing City, Southeast China | 119 | 44.8 | - | 35.6 | 0.08 | 0.31 | 97 | 11.2 | Hu et al., 2018 |
| Xianyang City, Northwest China | 190.54 | 25.52 | 25.33 | 39.44 | 0.13 | 0.78 | 55.36 | 8.35 | Wang et al., 2018 |
| Lisbon City, Portugal | 86 | 31.5 | 93.7 | 66.1 | - | <LOD | 63.6 | - | Bechet et al., 2017 |
| Nantes City, France | 86.4 | 42.3 | 30.1 | 60.6 | - | 2.1 | 33.3 | - | Bechet et al., 2017 |
| Changqun City Northeast China | 83.89 | 22.66 | 25.79 | 31.66 |  | 0.1347 | 55.19 | - | Liu et al., 2014 |
| Jilin City, Northeast China | 135.14 | 26.41 | 23.07 | 30.84 | - | 0.1434 | 65.65 | - | Liu et al., 2014 |
| Siping City, Northeast China | 91.98 | 30.41 | 20.7 | 28.51 | - | 0.2065 | 49.66 | - | Liu et al., 2014 |
| Songyuan City, Northeast China | 86.64 | 14.02 | 20.75 | 22.7 | - | 0.1701 | 37.04 | - | Liu et al., 2014 |
| Beijing, China | 78.03 | 26.78 | 21.22 | 22.64 | 0.13 | 0.2 | 58.15 | 7.99 | Zou et al.,2018 |
