# Supplementary material for: Ecological risk assessment and source identification of heavy metal pollution in vegetable bases of Urumqi, China, using the positive matrix factorization (PMF) method
Source: PLoS One. 2020 Apr 13;15(4):e0230191. doi: 10.1371/journal.pone.0230191 (PMC7153853; doi:10.1371/journal.pone.0230191)
Supplement: Attachment — Submitted filename: review plos one.doc [file pone.0230191.s002.doc]

The manuscript represents good quality of work. However, there are certain shortcomings which are to be incorporated to get the manuscript published in such reputed journal.

- First line of **introduction** is a repeat of same as in **abstract**. So it can be deleted in **Introduction**.
- The results of pH, Soil texture and SOM mentioned in **Materials and Methods** (Section 2.3) should be presented in **Results and Discussion** section and instead the methodology part should be made clearer. For example, How was SOM measured?
- The statement : ‘The results from several former researchers studies were listed in the Table S1’ . Table S1 could not be seen. If it is supplementary, it should be opened via link.
- In Line 390, 432, 471, 514, there is mention of Table.3, but the table does not exist in manuscript.
- In figure captions “5th and 95th and 5th and 95th percentiles of all data”, correct it as ‘5th and 95th percentiles of all data’,
- Although results are well written but discussion needs improvement. If other studies are mentioned in table S1 (which is missing here), these studies are also to be correlated to the present work.

Accepted only after minor revisions
