# Supplementary material for: Ecological risk assessment and source identification of heavy metal pollution in vegetable bases of Urumqi, China, using the positive matrix factorization (PMF) method
Source: PLoS One. 2020 Apr 13;15(4):e0230191. doi: 10.1371/journal.pone.0230191 (PMC7153853; doi:10.1371/journal.pone.0230191)
Supplement: Attachment — Submitted filename: Response to Reviewers.doc [file pone.0230191.s003.doc]

Response to Reviewers

- Reviewer #1:

1. Please avoid using first person point-of-view terms (e.g., we, our, my, etc.)

**Response:** Thank you for your comments and suggestions. We had improved the expression of this paper, please see the revised paper for details.

2. Please rewrite Key words to highly summarize the main job of your article.

**Response:** Thank you for your suggestions. We had revised Key words of this paper, please see the revised paper for details (which are highlighted in blue).

2. In lines 104-106, please check the terminology of source apportionment out twice.

**Response:** Thank you for your advice. We checked the lines 104-106 of this paper and we couldn't find any problem as you said in this sentence (To control and prevent heavy metal pollution, the source identification and apportionment are very important, and the selection of a proper and effective model is essential for accurate results)

3. In the Introduction section, you only mentioned the importance of receptor model (PMF). However, there is no implication about ecological risk assessment, which is very important in this work. Please involve it to your Introduction.

**Response:** Thank you for your suggestions. We had revised introductions of this paper, please see the revised paper for details (which are highlighted in blue).

5. Please avoid irregular writing format like lines 145, 178 and 195, etc.

**Response:** Thank you for pointing out the mistake. We had revised and please see the revised paper for details (which are highlighted in blue).

6. Why was the even lower value larger than the low value in lines 347-348?

**Response:** Thank you very much for your comments. The RI values of the Liushihu village was lower than other town or village, because this village far from the industrial area and have a less population, Furthmore, groundwater (in this area groundwater less polluted than surface water) was the main source for irrigation of this village.

7. In lines 425, authors said that the coefficient of variation of As was the largest value among the eight heavy metals, please discuss it in depth.

**Response:** Thank you for your suggestions.

8. The conclusions must be written in accordance with the objectives and with the results obtained. Please improve this part.

**Response:** We are extremely thankful your valuable comments that have greatly improved the clarity of this article.

Reviewer #2:

• First line of introduction is a repeat of same as in abstract. So it can be deleted in Introduction.

**Response:** Thank you for pointing out the mistake. It has now been corrected.

• The results of pH, Soil texture and SOM mentioned in Materials and Methods (Section 2.3) should be presented in Results and Discussion section and instead the methodology part should be made clearer. For example, How was SOM measured?

**Response:** Thanks a lot for this constructive comments and suggestions. The methods of the manuscript are refined as the reviewer commented. please see the revised paper for details (which are highlighted in red).

• The statement: ‘The results from several former researchers studies were listed in the Table S1’. Table S1 could not be seen. If it is supplementary, it should be opened via link.

**Response:** Thanks a lot for this valuable comment and pointing out the shortcoming. As reviewer suggested, the supplementary table take on opened via link.

• In Line 390, 432, 471, 514, there is mention of Table.3, but the table does not exist in manuscript.

**Response:** Thank you for pointing this mistake out. It has now been corrected. Please check the Line 390, 432, 471, 514 (which are highlighted in red).

• In figure captions “5th and 95th and 5th and 95th percentiles of all data”, correct it as ‘5th and 95th percentiles of all data’,

**Response:** Thank you for pointing out this mistake. It has been revised in figure caption list.

• Although results are well written but discussion needs improvement. If other studies are mentioned in table S1 (which is missing here), these studies are also to be correlated to the present work.

**Response:** we are very appreciated your valuable comments that have greatly improved the clarity of this article.

The editor’s suggestion and reviewer’s comments were really helpful and gave us a better perspective of our work. We hope that the revised manuscript would meet the requirement for publication.

Thanks again for your kindly help.

Sincerely yours,

Miradil Kurban, Balati Maihemuti, Ezzatgul Wali and Tuerxun Tuerhong

^*^Corresponding author: Balati Maihemuti

Address: College of Resource and Environment Sciences Xinjiang University, 14 Shengli Road, Tianshan Region, Urumqi 830046, People’s Republic of China

E-mail addresses: [bmaihemuti@xju.edu.cn](mailto:bmaihemuti@xju.edu.cn)
