# Supplementary material for: Ecological risk assessment and source identification of heavy metal pollution in vegetable bases of Urumqi, China, using the positive matrix factorization (PMF) method
Source: PLoS One. 2020 Apr 13;15(4):e0230191. doi: 10.1371/journal.pone.0230191 (PMC7153853; doi:10.1371/journal.pone.0230191)
Supplement: Attachment — Submitted filename: Response to Reviewers-2.doc [file pone.0230191.s004.doc]

Response to ACADEMIC EDITOR

ACADEMIC EDITOR: I agree with the several very serious concerns of the Reviewer 1 who is in full agreement with the critics to this manuscript. Kindly revise the manuscript thoroughly.

**Reply from Authors:** We at the first place are very much thankful to you for giving us an opportunity to revise our manuscript according to the comments of the reviewers. Also we wish to express our heartfelt thanks to you for your systematic and scientific arrangement of peer review process. We would also like to thank the current three reviewers for their time spent in reviewing our manuscript and for their constructive comments. We have addressed all the points raised by the reviewers and also consequently revised the manuscript (the highlighted parts) as per the reviewers’ comments. The revision also has been proofread by native English speaker with the language editing service of American journal experts. Definitely based on their comments, we feel that the scientific quality of manuscript has been considerably improved to be selected for publication in PLOS ONE. We have great pleasure in submitting our revision with the response to the reviewers’ comments for your kind perusal and consideration.

Response to Reviewers

Reviewer #1: Most of the comments that I sent previously were not answered/considered. Some problems still laid on this work, the first-person point-of-view terms are still used in this article, the key words are still cannot stand for this work, and the discussions are still in poor situation. Therefore, this article cannot be considered by this journal currently.

**Reply from Authors:** Thank you very much for your time spent in reviewing our manuscript and for your constructive comments at the first place. We have addressed all the points you raised, as well as we improved the discussions part of this MS and please see the revised paper for details (which are highlighted).

Reviewer #3: The research work presented in manuscript is important considering in increase in Pollution and thus heavy metals in biosphere. Though, some minor revisions are still required for the manuscript to be ready for publication.
• Use of Pronouns should be avoided in manuscript.
• Language needs improvement in the entire manuscript. Some sentences are incomplete, while others are not grammatically correct.
• Are you sure this is the first report on ecological risk associated with heavy metals in the vegetable bases, as mentioned in introduction? Or this is the first such report for the particular area?
• Probably you used SPSS 19.0 and not SPPS 19.0 as mentioned in line 242, please check.
• The sentence “The low (approx. 0-150) and even lower (approx. 150-300) RI values…….” Is still not clear. It can be rewritten as “in Liushihu village, where the density of human 351 activities and the amount of transportation were relatively low, groundwater was the 352 main source for irrigation and it was located far from urban and industrial areas, RI values exhibited light and moderate risk. Please modify.

Thank you very much for your encouraging and positive comments about our manuscript. We have addressed all the points you raised and also consequently revised the manuscript (the highlighted parts). Following are the detail reply to your concern.

• Use of Pronouns should be avoided in manuscript.
• Language needs improvement in the entire manuscript. Some sentences are incomplete, while others are not grammatically correct.
The revision has been proofread by native English speaker with the language editing service of American journal experts.

• Are you sure this is the first report on ecological risk associated with heavy metals in the vegetable bases, as mentioned in introduction? Or this is the first such report for the particular area?

We apologize for not being clear about this. Our research team has been conducting environmental pollution studies in this area (especially soil and groundwater pollution). some of our research findings has published on peer-reviewed Journal (Sawut, R., Kasim, N., Maihemuti, B., Hu, L., Abliz, A., Abdujappar, A., & Kurban, M. (2018). Pollution characteristics and health risk assessment of heavy metals in the vegetable bases of northwest China. Science of The Total Environment, 642, 864–878. doi:10.1016/j.scitotenv.2018.06.034). In our previous study only focused on analyzing the health risk assessment and pollution characteristics of six heavy metals on this vegetable basis but did not specifically perform heavy metal source apportionment. In this study, we use the geo-accumulation index (Igeo), potential ecological risk index (RI) to evaluate the present pollution states and potential risks and use the positive matrix factorization (PMF) method to fully identify the possible different pollution sources and relative contributions of the eight heavy metals.

Probably you used SPSS 19.0 and not SPPS 19.0 as mentioned in line 242, please check.
Thank you for pointing out the mistake. We had revised and please see the revised paper for details (which are highlighted).

The sentence “The low (approx. 0-150) and even lower (approx. 150-300) RI values…….” Is still not clear. It can be rewritten as “in Liushihu village, where the density of human 351 activities and the amount of transportation were relatively low, groundwater was the 352 main source for irrigation and it was located far from urban and industrial areas, RI values exhibited light and moderate risk. Please modify.

We thank the reviewer for the constructive criticism. We had revised and please see the revised paper for details (which are highlighted).

We thank all reviewers for their constructive comments on this manuscript. The editor’s suggestion and reviewer’s comments were very helpful and gave us a better perspective of our work. We hope that the revised manuscript would meet the requirement for publication.

Thanks again for your kindly help.

Sincerely yours,

Miradil Kurban, Balati Maihemuti, Ezzatgul Wali and Tuerxun Tuerhong

^*^Corresponding author: Balati Maihemuti

Address: College of Resource and Environment Sciences Xinjiang University, 14 Shengli Road, Tianshan Region, Urumqi 830046, People’s Republic of China

E-mail addresses: [bmaihemuti@xju.edu.cn](mailto:bmaihemuti@xju.edu.cn)
